# COVID-19 Misinformation Online and Health Literacy: A Brief Overview

**DOI:** 10.3390/ijerph18158091

**Published:** 2021-07-30

**Authors:** Salman Bin Naeem, Maged N. Kamel Boulos

**Affiliations:** 1 Department of Library & Information Science, The Islamia University of Bahawalpur, Bahawalpur 63100, Pakistan; salman.naeem@iub.edu.pk; 2 Information Management School, Sun Yat-sen University, Guangzhou 510006, China

**Keywords:** COVID-19, infodemic, misinformation, disinformation, social media, health literacy, digital health literacy

## Abstract

Low digital health literacy affects large percentages of populations around the world and is a direct contributor to the spread of COVID-19-related online misinformation (together with bots). The ease and ‘viral’ nature of social media sharing further complicate the situation. This paper provides a quick overview of the magnitude of the problem of COVID-19 misinformation on social media, its devastating effects, and its intricate relation to digital health literacy. The main strategies, methods and services that can be used to detect and prevent the spread of COVID-19 misinformation, including machine learning-based approaches, health literacy guidelines, checklists, mythbusters and fact-checkers, are then briefly reviewed. Given the complexity of the COVID-19 infodemic, it is very unlikely that any of these approaches or tools will be fully effective alone in stopping the spread of COVID-19 misinformation. Instead, a mixed, synergistic approach, combining the best of these strategies, methods, and services together, is highly recommended in tackling online health misinformation, and mitigating its negative effects in COVID-19 and future pandemics. Furthermore, techniques and tools should ideally focus on evaluating both the message (information content) and the messenger (information author/source) and not just rely on assessing the latter as a quick and easy proxy for the trustworthiness and truthfulness of the former. Surveying and improving population digital health literacy levels are also essential for future infodemic preparedness.

## 1. Introduction

The COVID-19 pandemic has been accompanied by an explosion of inaccurate information about the disease, making it difficult for the general public to make informed decisions. The exact scale of spread of the excessive amount of online COVID-19 misinformation is still unknown but is nevertheless a cause of high concern. Misinformation can be broadly defined as “cases in which people’s beliefs about factual matters are not supported by clear evidence and expert opinion” [1]. It is false information being spread regardless of intent to mislead, while disinformation is false information being spread with bad intent, to deliberately deceive. The Director-General of the World Health Organization (WHO) termed the COVID-19 misinformation situation as an ‘infodemic’ (i.e., misinformation epidemic or misinformation pandemic) swarming with conspiracy theories, propaganda, and unproven scientific claims regarding the diagnosis, treatment, and prevention of the disease [2,3]. This infodemic has made reliable information harder to find and discern, and allowed rumours to spread more quickly, putting public health at risk by making it difficult to implement effective preventive measures [3].

## 2. COVID-19-Related Misinformation on Social Media

Social media outlets, such as Facebook, Twitter, YouTube, etc., emerged as major information seeking and sharing channels during the pandemic. As a result, the use of social media platforms increased by 20–87% around the world [4]. During health crises, access to reliable information sources and services becomes critical to enable the general public to take part in healthcare and preventive decisions. However, the abundance of health information on social media without any comprehensive checks makes it difficult for the general public to identify accurate information, thus impeding effective public health response [5].

Fondazione Bruno Kessler analysed 112 million posts shared on social media related to COVID-19, and concluded that more than 40% of posts contained information from unreliable sources, while 42% of posts were circulated on social media platforms through bots [4]. Lee et al. reported that 67.78% of adults have been exposed to COVID-19-related misinformation through social networking services or instant messaging [6]. In another study, an analysis of 1225 fake news stories about COVID-19 from January to April 2020 showed that social media accounted for spreading half or 50.5% of the stories [5]. Content spreading misinformation about vaccines gained 4.5 billion views on social media in just one month (March to April 2020) [7]. Several studies indicated that the infodemic of misleading information during COVID-19 is a threat to the COVID-19 vaccination campaign [8].

A comprehensive systematic review of studies published before March 2019 on the prevalence of health misinformation on social media reported widespread misinformation in six areas, namely (i) vaccines, (ii) drugs or smoking, (iii) non-communicable diseases, (iv) pandemics, (v) eating disorders, and (vi) medical treatments. Twitter was the leading source spreading health-related misinformation [9]. Naeem and colleagues classified the most commonly identified fake news about COVID-19 into three main types: (i) false claims, such as “coronavirus can be transmitted through houseflies or mosquito bites”, (ii) conspiracy theories, such as the ones about 5G (fifth generation technology standard for broadband cellular phone networks) and coronavirus, and (iv) pseudoscientific health therapies, such as “colloidal silver solution can help with coronavirus” [5]. A content analysis of a sample of 233 tweets identified 81 tweets (34.8%) falsely claiming that COVID-19 and 5G were linked, 75 tweets (32.2%) criticising the conspiracy theory, and 77 general tweets (33%) not giving any personal remarks or opinions. Surprisingly, 152 (65.2%) of tweets were from people who were non-conspiracy-theory supporters [10].

Digital communications, particularly on social media platforms, catalyse the rapid spread of misinformation, threatening public health [3,10]. Bridgman et al. examined 0.6 million tweets on COVID-19 and found that the use of social media was positively associated with misperceptions regarding basic COVID-19 facts. The opposite was true for news media. The study also revealed that comparatively more misleading content related to COVID-19 circulated on Twitter as compared to traditional news media [11]. In the same vein, Su concluded that social media use is significantly associated with misinformation beliefs related to COVID-19 [12].

Evidence from several studies showed that misleading information and conspiracy theories spread more rapidly when the flow of factual information is slow, and when people’s trust in the information sources and services available to them is also low, or when credible information is hard to come by [13,14,15]. Tworek argues that “communications in a public health crisis are as crucial as medical intervention. In fact, communications policies ARE a medical intervention” [16]. Effective health communication plays a critical role in speeding up the flow of factual information and building trust among people regarding health information sources and services.

The United Nations Development Programme has directed governments across the world to “step up to lead the fight against a growing tide of false, inflammatory and misleading information that threatens to worsen the already severe impacts of the virus” [17]. There is a broad commitment among researchers, healthcare professionals, information professionals, IT experts, policymakers, and social media experts to prevent the spread of misinformation.

## 3. Health Literacy and Healthcare Outcomes

Health literacy has received significant attention since the year 2000, when the WHO stated that a low level of public health literacy is a serious concern for public health, and stressed the need to improve public health literacy to minimise inequities in health services [18]. Health literacy covers “the ability to access, comprehend, evaluate and communicate information to promote, maintain and improve health in a variety of settings across the life course” [19]. Health literacy has been defined by WHO as the individual features and societal resources required for the public and individuals to obtain, recognise, and evaluate information and services in order to make appropriate decisions about health. Health literacy allows and encourages the public to contribute to, and improve, their healthcare and well-being. People possessing good health literacy usually have more skills to manage their health in a better way compared to those with low health literacy skills. The WHO considers the level of people’s health literacy an indicator that can be used in assessing the health status of the population [20].

Health literacy has three main pillars, namely (i) the capacity to obtain health information (where to find help), (ii) the ability to (properly) understand the information gathered, and (iii) the ability to (properly) apply health information [21]. The Australian Commission on Safety and Quality in Health Care (ACSQHC) describes two main components of health literacy: (i) individual health literacy, comprising the individual skills and ability to find, understand and use health-related information; for example, to be able to navigate and use health/care systems, and (ii) the health literacy environment, covering components related to a health system, such as policies, materials and procedures that affect the way people engage with the system [22].

Significant amounts of health-related information and myths about COVID-19 are not sufficiently simple or clear to easily comprehend or debunk by the average layperson. The pandemic and associated infodemic have brought unprecedented challenges to public health, affecting the public throughout the world in terms of socio-psychological and economic hardships, and stressing international relations (e.g., by originally naming COVID-19 variants after the places or countries where they were first reported; the WHO now recommends using Greek letters to neutrally refer to these variants). Digital technologies have amplified misinformation, but could also hold the solution.

The World Health Organization is a strong promoter of digital health literacy. With ‘digital’ added to the formula, we can anticipate wider accessibility of reliable health information, improved healthcare quality and reduced healthcare costs [23]. Digital health literacy is the ability to seek, find, understand, and appraise health information using information and communication technologies (ICTs) to address or solve a healthcare problem [20,24,25].

### Prevalence of Low Health Literacy

Low health literacy is very common among people across the world. According to a large-scale national survey, more than 1 in every 3 adults in America have low health literacy [26]. Only 8.8% of people were health literate in China in 2012, leading the National Health Commission of China to issue a new strategic plan in the year 2014 to increase the health literacy of the population to 20% by 2020 [27]. In the UK, 4 in 10 adults struggle to understand health information that is meant for the public, and more than 6 in 10 adults struggle with health-related information that includes numbers and statistics [28,29].

The evidence also shows that people with a low level of health literacy skills are more likely than those people with a greater level of health literacy skills to report poor health [30]. COVID-19 impacted people with inadequate health literacy more badly and more frequently as compared to people with proficient health literacy levels, due to the inability of the former to properly understand and follow health-related recommendations. They find it difficult to locate healthcare providers and healthcare services, share their medical condition and history with healthcare providers, seek preventive healthcare, understand directions on medicines (prescriptions), and recognise the connection between risky behaviours and health [31].

A large-scale systematic review covering 111 published studies that measured health literacy and its association with health outcomes concluded that low health literacy is a contributor to compromised health outcomes and inadequate use of healthcare services, resulting in greater use of emergency services, increased hospitalisations, lower vaccination rates, improper medication-taking, inadequate ability to interpret prescriptions and health messages, and higher mortality rates among elderly persons. People with poor health literacy skills are at higher risk of poor health outcomes and unhealthy behaviours [24].

The literature is abundant with studies covering these negative consequences; for example, research linking low health literacy to a lower engagement with health services [32] and healthcare providers [33], to inadequate use of preventive health services [34], to poor health status and increased hospital re-admission rates [35], to a lack of understanding of medication instructions [36], to a lower quality of life in specific medical conditions [37], and to poor ability to self-manage health [38]. Low health literacy is also strongly correlated with health inequalities [39].

Health literacy is context-dependent. This means people with generally acceptable health literacy skills can still face health literacy challenges in some contexts, such as when they are not familiar with some complex medical jargon, or in certain health conditions requiring complicated self-care. Abdulai et al. assessed digital literacy levels among lay consumers of online COVID-19 information in Ghana, a low-income country, using a survey based on the eHealth Literacy Scale (eHEALS). Not only did the majority of respondents in their study exhibit poor ability to find COVID-19 information online, but they also demonstrated relatively low skills in differentiating scientific from unscientific information on the Internet. Gender, age, frequency of searching the Internet for COVID-19 information and for educational purposes, and having adequate or poor knowledge of COVID-19 were predictive of digital literacy levels [40]. Socioeconomic status, inadequate education, limited English proficiency, older age, and being from a diverse background are among the main factors linked to low health literacy [41,42,43,44]. In another survey of South Korean adults in 2020, Lee et al. found that COVID-19 misinformation exposure was associated with misinformation belief, and that misinformation belief was linked to fewer preventive behaviours [6].

We live in a highly digitised world where every piece of information available online is potentially subject to authenticity and validity checks. Thus, we must recognise and promote e-health and media literacies as fundamental skills, just as writing and reading are. Digital health and media literacies should also be promoted as lifelong-needed competencies that, once acquired, could be useful in any health-related context. Only then will we be able to empower people to better identify and protect themselves from misinformation and to make correctly informed decisions about their health [45]. Many studies recommend developing good digital health literacy skills among the public as a way forward to critically (self-)appraise information and prevent the spread of misinformation during pandemic times [40,46]. Health literacy and numeracy skills are two fundamental skill sets that should be promoted among the public in order to ensure better healthcare outcomes [30].

## 4. Strategies and Methods to Detect and Prevent the Spread of Misinformation

To prevent the spread of COVID-19 misinformation, a number of guidelines, checklists, resources, fact-checking tools, mythbusters, digital health literacy training instructions, and long-term strategies have been proposed, developed and/or implemented. These misinformation mitigation attempts have mainly been made by computer scientists, data science experts, information professionals, researchers, government agencies, and professional associations. Computer scientists developed fact-checking tools, applications and resources using artificial intelligence-based approaches, including machine learning, while information professionals, social and behavioural science researchers, and professional associations created guidelines and checklists to help the general public recognise fake news.

Given the complexity of the infodemic, no approach has yet managed to fully automate the challenge of fake news detection. According to the Fake News Challenge Stance-Detection Task’s creators, ‘truth labelling’ is very difficult in practice. They proposed developing a dedicated tool for that purpose, preferring a reliable semi-automated approach over a fully-automated system in order to achieve a greater percentage of accuracy in detecting fake news [47]. Although, fully automated systems have received some success in recognising information validity, their accuracy has not (yet) reached an acceptable level. In one study published in late 2019, the best performing system achieved a FEVER (Fact Extraction and VERification) score of 64.21% [48]. The rapidly changing COVID-19 information landscape and the associated relatively “novel” pandemic language make currently used (pre-pandemic) misinformation recognition datasets and models ineffective for debunking COVID-19 misinformation [49].

Daniel J. Levitin, a neuroscientist, said that “what looks like (and reads like) the truth may be riddled with lies if you look more closely”. He identified “four tricky ways that fake news can fool you”. These include (i) “lies are tucked in among truths”, (ii) “websites masquerade under misleading names”, (iii) “numbers are given without context”, and (iv) “claims rest on false sources”. He recommended that the task of figuring out the truth and discarding fake news falls on us all, and requires critical thinking and time. It also requires us to keep updating our knowledge as information continues to evolve very rapidly [50].

### 4.1. Machine Learning-Based Approaches

Several tools, approaches, and models have been reported in the literature that can flag COVID-19 related misinformation as false information. For example, Hossain et al. [49] introduced COVIDLies, a tweet debunking dataset annotated by experts, containing COVID-19 misinformation accompanied by tweets that ‘agree’, ‘disagree’, or express ‘no stance’ for each piece of misinformation. Abdelminaam and colleagues proposed CoAID-DEEP (COVID-19 heAlthcare mIsinformation Dataset), a machine learning and deep learning system to automate the identification of COVID-19-related fake news [51]. Kolluri and Murthy introduced CoVerifi, a Web application that attempts to appraise the credibility of news based on human feedback and the power of a machine learning-based approach. It provides a multi-channel check for news, using user feedback to detect fake news that have failed to be recognised by Web-based fact-checking tools and vice versa [52]. Khanday et al. argue that machine learning and deep learning can replace humans in providing accurate results regarding fake news detection [53], but it is obvious that such a bold claim cannot be universally applied in every situation.

### 4.2. Health Literacy Guidelines, Checklists, Mythbusters and Fact-Checkers

The International Federation of Library Associations’ (IFLA) guidelines are among the most widely accepted and used fake news detection checklists available today. It comprises eight steps: (i) “consider the source”, (ii) “check the author”, (iii) “check the date”, (iv) “check your biases”, (v) “read beyond”, (vi) “seek supporting sources”, (vii) ask “is it a joke?”, and (viii) “ask the experts” [54].

CRAAP (Currency, Relevance, Authority, Accuracy, and Purpose) is another useful checklist for evaluating online information resources, created by the Meriam Library, California State University. It focuses on whether an information resource is ‘current’ and up-to-date, ‘relevant’, ‘accurate’, and ‘telling the truth’, and directs information evaluators to seek answers to questions such as who is the ‘author’ (who wrote or compiled the resource under investigation), and what is the resource’s ‘purpose’ or why was it created [55].

The World Economic Forum’s three-step guidance on “how to read the news like a scientist and avoid the COVID-19 infodemic” is quite handy for debunking misinformation. The three steps are: (i) “embracing uncertainty responsibly”, (ii) “asking where is the information coming from?” and (iii) “determining who is backing up the claim” [56].

Llewellyn proposed a contact-tracing-like technique to identify COVID-19 misinformation. As with coronavirus itself, we should think about “contact tracing” the message and its sender(s): “who sent or shared the information”, “what is the original source”, and “how do I know it to be true?” [57]. Mantas compiled a six-step kit after evaluating various fact-checking organisations. These steps include: (1) “stop, breathe—before sharing” (2) “check the source”, (3) “trust scientists before politicians”, (4) “beware your emotions”, (5) “use (adequate) tools to help verify images and videos”, and (6) “know what you don’t know” [58].

Mheidly and Fares proposed an “infodemic response checklist” to help overcome the challenges posed by the infodemic. Their checklist recommends a number of mitigatory actions, including (i) offering more exposure or ‘airtime’ for medical professionals and scientists, (ii) verifying the social media accounts of scientists, medical professionals and public health officials, (iii) sharing scientific evidence, (iv) promoting the Web sites of public health organisations via search engines, (v) monitoring engagement on social media platforms, (vi) developing infographics and educational material, (vii) addressing minorities, racial, and ethnic differences, (viii) adopting empathy in social media communication, (ix) promoting dialogue and sharing of personal experiences, (x) improving coping skills against mental health stressors, and (xi) boosting investment in health communication research [59].

The United Nations Educational, Scientific and Cultural Organization--UNESCO ‘MIL CLICKS’ social media initiative for promoting media and information literacy is helping people acquire key skills related to media and information literacy in their normal day-to-day use of the Internet and social media. ‘MIL CLICKS’ is an acronym that stands for ‘Media and Information Literacy: Critical-thinking, Creativity, Literacy, Intercultural, Citizenship, Knowledge and Sustainability’ [60].

Conard developed a health literacy instructional model combining five domains of literacy (knowledge, numeracy, navigation, communication, and decision making) with three components (emotional, behavioural, and educational) to help build health literacy skills. The model presents a framework for addressing the emotional state of the individual in the first stage, with a second stage offering strategies that aim at strengthening the engagement and commitment of the person using a behavioural approach, and a final phase delivering an educational solution [61].

The iHeal.eu project (November 2018–April 2021), co-funded by the European Commission, is another successful programme for up-skilling older EU citizens (aged 50+) in digital health literacy to prevent marginalisation and exclusion. Project outputs included (i) a transnational digital health literacy ecosystem map and methodological framework, (ii) a curriculum, with storyboards and content, (iii) an interactive Web platform, learning environment, applications, and digital learning tools, and (iv) a training adaptation toolkit [62].

Besides the roles played by the aforementioned checklists and guidelines in tackling the COVID-19 infodemic, mythbusters have contributed significantly to flagging COVID-19 related misinformation and promoting factual information. iHealthFacts.ie is a Web-based resource where the public can easily and quickly check the authenticity of common health claims circulated on social media [63]. It is a useful resource, helping people to think critically about health claims and make informed decisions about their health [64]. Other examples of mythbusters are provided in Table 1.

Several Web-based fact-checking tools are also available that allow people to check the authenticity of a specific claim. The majority of reputable fact-checking sites subscribe to codes of practice and standards that explicitly call for neutrality and transparency, such as the International Fact-Checking Network’s (IFCN) code of principles [70]. Together with the very closely related online mythbusters, these fact-checkers are helping combat the spread of COVID-19-related misinformation. Examples of these fact-checking tools are provided in Table 2.

## 5. Discussion and Recommendations

Low digital health literacy affects large percentages of populations around the world. It is a direct contributor to the spread of COVID-19 misinformation (e.g., via sharing on social media) as result of the affected populations’ inability to recognise falsehoods and their high vulnerability to deception by fake news (bots also contribute to the spread of online misinformation). The ‘viral’ nature of social media sharing further complicates the situation [76].

Given the complexity of the COVID-19 infodemic and the high prevalence of low digital health literacy among populations, it is very unlikely that any of the above-mentioned strategies, methods, and services (e.g., machine learning-based approaches, health literacy guidelines, and fact-checkers) will be fully effective *alone* in stopping the spread of COVID-19 misinformation. Banerjee and Meena offer a useful summary of the different ways in which digital technology and (social) media platforms can be used during health emergencies, such as the COVID-19 pandemic, to aid the general public [77].

Interestingly, Zhao et al. found a correlation between people’s preferences for particular media outlets when seeking health-related information and their behaviours during the pandemic in the USA. For example, people who considered Fox News a more trustworthy information source compared to CNN (Cable News Network) demonstrated a higher number of risky behaviours and less preventive behaviours in relation to COVID-19 (e.g., not wearing face masks). People’s behavioural responses to the pandemic were clearly divided along media bias lines. News outlets should therefore be made accountable, and their unhelpful partisan stance should be minimised during health emergencies [78].

Medical/health journalists reporting medical research in mainstream news media often unwittingly contribute to online health misinformation through bad reporting practices and distorted translation of research findings in their news reports, e.g., misinterpreting or exaggerating findings and other forms of inaccurate and/or incomplete reporting, resulting in public confusion. However, well trained, ethical medical/health journalists can play an important role in combatting misinformation.

In fact, in his July 2021 ‘*Advisory on Building a Healthy Information Environment—Confronting Health Misinformation*’, the US Surgeon General calls for media, science, digital, data and health literacy programmes and training to be provided for journalists, as well as health practitioners, librarians and others, as part of longer-term efforts to build resilience against health misinformation. He also urges the implementation of product design and policy changes on technology platforms to slow the spread of misinformation, in addition to expanding research that deepens our understanding of health misinformation and the best ways of tackling it [79].

Health planners and public health professionals responsible for ensuring future infodemic preparedness should comprehensively survey the digital health literacy levels of their target populations to determine the best strategies for improving these levels, namely well-targeted community outreach and awareness-raising interventions, including well-tailored education and training programmes that closely match the health literacy levels and specific needs of target audiences [20]. A number of (mostly complementary) scales and tools exist for measuring (digital) health literacy, e.g., eHEALS [40,80], NVS (Newest Vital Sign) [79,81], eHLA (eHealth Literacy Assessment Toolkit) [82], etc. Some (but not all) of these tools are available in multiple languages. Using two or more of these tools in conjunction is often required in order to achieve a more complete and accurate picture of the surveyed population’s digital health literacy levels.

Improving people’s digital health will enable them to more (pro)actively and effectively participate in the healthcare system. There is a pressing need to raise awareness among the general public that they should only seek information from valid, trusted sources [83], or as Charlie Baker, the Governor of Massachusetts, rightly suggested that “Everybody needs to get their news from legitimate places, not from their friend’s friend’s friend’s friend” [84].

However, Kamel Boulos argues that it is hard to be objective and to remain so at all times, calling for a comprehensive evaluation of *both* ‘the message’ (information content) and ‘the messenger’ (information author/source), rather than fully relying on the latter as (an-easy-to-establish) proxy for the trustworthiness and truthfulness of an information resource. All systems of peer review and fact-checking are inherently flawed to some extent and prone to subjectivity and influence by conflicting interests and agendas, including political ones. Having information spread or published by a reputable academic belonging to a prestigious institution, or an author with recognised qualifications, or a prestigious-scientific-award winner, or a high-impact-factor scholarly journal does not always guarantee quality (and does not disapprove it either). Consumers of online information should always take ‘recognised qualifications’, ‘prestigious institutions’, ‘fact-checking sites’, ‘peer review’, ‘reputable journals’ (and their impact factors), etc., with a grain of salt. These information-source attributes are all relative rather than absolute, and are always more helpful and useful when considered together, combined rather than individually, and when considered alongside other equally, if not more, important information quality criteria that focus on the message rather than the messenger [85].

## 6. Conclusions

Low digital health literacy affects large percentages of populations around the world and is a direct contributor to the spread of COVID-19-related online misinformation and to its devastating effects. The ease and ‘viral’ nature of social media sharing further complicate the situation. A number of strategies, methods, and services exist that can be used to detect and prevent the spread of COVID-19 misinformation, including machine learning-based approaches, health literacy guidelines, checklists, mythbusters, and fact-checkers. However, given the complexity of the COVID-19 infodemic, it is very unlikely that any of these approaches or tools will be fully successful alone in combatting COVID-19 misinformation. Instead, a mixed, synergistic approach, combining the best of these strategies, methods, and services together, is essential as the most effective way forward to tackle online health misinformation, and mitigate its negative effects in COVID-19 and future pandemics. Furthermore, to achieve the best results possible, techniques, and tools should focus on evaluating *both* the message (information content) and the messenger (information author/publisher/source), and not just rely on assessing the latter as a quick and easy proxy for the trustworthiness and truthfulness of the former. Surveying and improving population digital health literacy levels is also key to future infodemic preparedness.

## Figures and Tables

**Table 1:** Examples of online COVID-19 mythbusters.

Service Name	Hosting Organisation and Web Address
The WHO Mythbuster	World Health Organization [65]
Australia COVID-19 Mythbuster	Australian Government [66]
Canada Corona Virus Misinformation Watch	Ryerson Social Media Lab at Ted Rogers School of Management, Ryerson University, Canada [67]
TAG Mythbusters	Treatment Action Group, a US charity in New York, NY [68]
Pakistan National Command and Control Centre Mythbuster	Government of Pakistan [69]

**Table 2:** Examples of online COVID-19 fact-checkers.

Service Name	Hosting Organisation and Web Address
ClaimBuster Fact Checker	The University of Texas at Arlington, USA [71]
Google Fact Check Tools/Explorer	Google [72]
FactCheck.org	The Annenberg Public Policy Center of the University of Pennsylvania, USA [73]
AFP Fact Check	Agence France-Presse, an international news agency headquartered in Paris, France [74]
BBC News Reality Check	The British Broadcasting Corporation, the national broadcaster of the United Kingdom [75]

## Data Availability

No new data were created or analysed in this study. Data sharing is not applicable to this article.
